# Prevalence of Voluntary Medical Male Circumcision for HIV Infection Prevention — Chókwè District, Mozambique, 2014–2019

**DOI:** 10.15585/mmwr.mm7026a2

**Published:** 2021-07-02

**Authors:** Jonas Z. Hines, Ricardo Thompson, Carlos Toledo, Robert Nelson, Isabelle Casavant, Sherri Pals, Marcos Canda, Juvencio Bonzela, Alicia Jaramillo, Judite Cardoso, Dawud Ujamaa, Stelio Tamele, Victor Chivurre, Inacio Malimane, Ishani Pathmanathan, Kristen Heitzinger, Stanley Wei, Aleny Couto, Jotamo Come, Alfredo Vergara, Duncan MacKellar

**Affiliations:** ^1^Division of Global HIV & TB, Center for Global Health, CDC; ^2^Chókwè Health Research and Training Centre, National Institute of Health, Chókwè, Mozambique; ^3^Division of Global HIV and TB, Center for Global Health, CDC, Maputo, Mozambique; ^4^Jhpiego, Johns Hopkins University, Maputo, Mozambique; ^5^Chókwè District Public Health Directorate, Chókwè, Mozambique; ^6^Provincial Directorate of Public Health, Gaza, Mozambique; ^7^Mozambique Ministry of Health, Maputo, Mozambique.

Male circumcision is an important preventive strategy that confers lifelong partial protection (approximately 60% reduced risk) against heterosexually acquired HIV infection among males (*1*). In Mozambique, the prevalence of male circumcision was 51% when the voluntary medical male circumcision (VMMC) program began in 2009. The Mozambique Ministry of Health set a goal of 80% circumcision prevalence among males aged 10–49 years by 2019 (*2*). CDC analyzed data from five cross-sectional surveys of the Chókwè Health and Demographic Surveillance System (CHDSS) to evaluate progress toward the goal and guide ongoing needs for VMMC in Mozambique. During 2014–2019, circumcision prevalence among males aged 15–59 years increased 42%, from 50.1% to 73.5% (adjusted prevalence ratio [aPR] = 1.42). By 2019, circumcision prevalence among males aged 15–24 years was 90.2%, exceeding the national goal (*2*). However, circumcision prevalence among males in older age groups remained below 80%; prevalence was 62.7%, 54.5%, and 55.7% among males aged 25–34, 35–44, and 45–59 years, respectively. A multifaceted strategy addressing concerns about the safety of the procedure, cultural norms, and competing priorities that lead to lack of time could help overcome barriers to circumcision among males aged ≥25 years.

CHDSS catchment areas located in Gaza Province included all households in Chókwè town and seven rural villages, representing approximately 100,000 of 183,000 total Chókwè District residents of all ages and approximately 58,000 residents aged 15–59 years. HIV is hyperendemic in this area; in 2015, HIV prevalence among males aged 15–49 years was higher in Gaza Province (17.6%) than in all other provinces in Mozambique (3.3%–15.8%) (*3*). During the analysis period, circumcision by certified providers was routinely offered at Hospital Rural de Chókwè and via a mobile operating unit at four temporary (outreach) sites in Chókwè District. Services were regularly advertised through local radio stations and promoted by community leaders during public engagements. In addition, lay counselors encouraged circumcision during household-based HIV-testing services, and campaigns that included free transportation to circumcision sites were periodically conducted in public spaces (e.g., markets, workplaces, and schools) to create demand. During 2014–2019, a total of 19,201 males aged ≥10 years in Chókwè District underwent voluntary medical circumcision.^†^

Prevalences of male circumcision and HIV infection among males were assessed with five independent, annual cross-sectional surveys of approximately 13% (survey rounds R1 and R2) or 23% (survey rounds R3–R5) of randomly selected CHDSS catchment area households.^§^ All members of selected households aged 15–59 years were eligible to participate in an interview, which included each male’s self-reported circumcision status, reasons for not being circumcised, and intention to undergo circumcision in the next 12 months (if applicable). Females were asked about their beliefs about male circumcision and whether they had ever discussed circumcision with a male sex partner, friend, or family member. All participants provided written consent. After the interview, consenting participants received a rapid HIV test in accordance with the national testing algorithm and provided 1–2 mL of whole blood. During R1–R3, stored blood specimens from males with HIV infection were used to evaluate recency of HIV infection.^¶^

Male circumcision aPRs (adjusted for age group, residence of Chókwè town [urban] or a CHDSS village [rural], and marital status [single versus nonsingle**]) and differences in HIV prevalence and incidence between circumcised and uncircumcised males were analyzed using SAS (version 9.3; SAS Institute). Annualized HIV incidence and 95% confidence intervals (CIs) were calculated using R (version 3.5.2; R Foundation).^††^^,^^ §§^ All estimates were census-weighted by sex, age group, and geographic area (urban or rural). Because all selected household members aged 15–59 years were eligible for the surveys, confidence intervals were adjusted for household clustering. The protocol was approved by the local institutional review board and the National Committee for Bioethics in Health of Mozambique. This activity was reviewed by CDC and was conducted consistent with applicable federal law and CDC policy.^¶¶^

The number of participants during R1–R5 ranged from 3,034 to 5,089 (response rate of contacted residents was 64.2%–84.3%). Overall, males accounted for 30% of all participants. Among 5,904 male survey participants during R1–R5, 5,837 (98.9%) reported their circumcision status. During 2014–2019, prevalence of male circumcision increased 42%, from 50.1% during R1 to 73.5% during R5 (aPR = 1.42) (Table 1). The largest increases occurred among males who resided in rural areas (37.0% to 62.5%; aPR = 1.77) and males aged 15–24 years (58.4% to 90.2%; aPR = 1.47). The increase in circumcision prevalence from R1 to R5 was less pronounced among older age groups studied: 25–34 years (44.7% to 62.7%), 35–44 years (39.6% to 54.5%), and 45–59 years (42.1% to 55.7%). Single males and those residing in urban areas were more likely to be circumcised than were nonsingle males or those living in rural areas; differences in circumcision prevalence between males living in urban and rural areas decreased from R1 (aPR = 1.55) to R5 (aPR = 1.17) (Table 1).

**TABLE 1:** Male circumcision prevalence by sociodemographic characteristics and survey round — Chókwè Health Demographic Surveillance System, Chókwè District, Mozambique, 2014–2019

Characteristic	Round 1 (Apr 2014–Apr 2015) N = 1,109		Round 2 (May 2015–Jan 2016) N = 872		Round 3 (Mar–Dec 2016) N = 1,362		Round 4 (Mar–Nov 2017) N = 1,318		Round 5 (Apr 2018–Mar 2019) N = 1,176		Round 5 versus Round 1
	% (95% CI)	aPR* (95% CI)	% (95% CI)	aPR* (95% CI)	% (95% CI)	aPR* (95% CI)	% (95% CI)	aPR* (95% CI)	% (95% CI)	aPR* (95% CI)	aPR* (95% CI)
**All males**	**50.1 (46.8–53.6)**	**N/A**	**57.1 (53.4–61.1)**	**N/A**	**65.5 (62.4–68.7)**	**N/A**	**66.5 (63.4–69.8)**	**N/A**	**73.5 (70.6–76.6)**	**N/A**	**1.42 (1.33–1.52)**
**Age group, yrs**											
15–24	58.4 (54.0–63.2)	Ref	72.0 (67.5–76.8)	Ref	82.7 (79.8–85.7)	Ref	84.7 (81.9–87.6)	Ref	90.2 (88.0–92.4)	Ref	1.47 (1.36–1.60)
25–34	44.7 (38.3–52.2)	0.81 (0.68–0.97)	42.0 (34.5–51.2)	0.62 (0.51–0.77)	53.9 (46.8–62.2)	0.69 (0.59–0.81)	53.1 (45.0–62.6)	0.68 (0.57–0.82)	62.7 (55.6–70.6)	0.77 (0.69–0.87)	1.40 (1.16–1.70)
35–44	39.6 (32.0–49.1)	0.73 (0.58–0.93)	46.1 (36.9–57.5)	0.70 (0.55–0.88)	48.6 (40.3–58.5)	0.64 (0.52–0.78)	45.6 (37.2–55.8)	0.60 (0.48–0.75)	54.5 (45.8–64.7)	0.68 (0.57–0.82)	1.37 (1.04–1.80)
45–59	42.1 (34.2–51.7)	0.78 (0.62–0.99)	42.7 (33.8–53.9)	0.66 (0.51–0.84)	43.2 (35.6–52.4)	0.59 (0.47–0.72)	49.5 (41.2–59.3)	0.67 (0.55–0.81)	55.7 (47.1–66.0)	0.71 (0.59–0.85)	1.33 (1.02–1.73)
**Marital status**											
Nonsingle†	41.6 (37.3–46.4)	Ref	44.0 (38.9–49.8)	Ref	51.0 (46.2–56.4)	Ref	52.4 (47.5–57.9)	Ref	57.6 (52.6–63.0)	Ref	1.42 (1.31–1.53)
Single	59.8 (55.3–64.6)	1.12 (0.97–1.29)	70.1 (65.4–75.1)	1.18 (1.03–1.36)	76.7 (73.2–80.3)	1.14 (1.01–1.28)	80.1 (76.7–83.7)	1.12 (1.01–1.26)	87.5 (84.8–90.2)	1.08 (0.99–1.17)	1.48 (1.29–1.69)
**Residence** ^§^											
Rural	37.0 (33.1–41.3)	Ref	46.5 (42.2–51.2)	Ref	57.9 (54.4–61.6)	Ref	59.2 (55.7–63.0)	Ref	62.5 (58.6–66.7)	Ref	1.77 (1.58–1.99)
Urban	56.7 (52.4–61.5)	1.55 (1.36–1.76)	62.6 (57.5–68.0)	1.36 (1.21–1.52)	69.4 (65.2–73.9)	1.18 (1.10–1.26)	70.3 (65.9–74.9)	1.15 (1.08–1.23)	79.1 (75.3–83.2)	1.17 (1.11–1.23)	1.34 (1.24–1.45)

**Abbreviations:** aPR = adjusted prevalence ratio; CI = confidence interval; N/A = not applicable; Ref = referent group.

*Adjusted for age group, marital status, and urban or rural residence.

^†^ Nonsingle was a composite variable of married, union, divorced, separated, and widowed.

^§^ Rural indicates residence in one of seven district villages; urban indicates residence in Chókwè town.

Among males aged 25–59 years who participated in R5 (April 2018–March 2019), few (3.0%) who were circumcised had undergone the procedure during the previous year. A considerable proportion (44.7%) of uncircumcised males in this age group reported that they intended to undergo circumcision during the next year (Table 2); these males were less aware (70.5%) than were their circumcised counterparts (85.4%) that male circumcision provides partial protection against HIV infection (aPR = 1.21; 95% CI = 1.07–1.37). Common reasons for not undergoing circumcision included fear of complications (26.6%),*** not perceiving male circumcision as part of one’s culture (17.2%), and lack of time (17.0%). Nearly all females who participated during R5 (96.0%) agreed that males should be circumcised.^†††^

**TABLE 2:** Knowledge, attitudes, and beliefs related to circumcision among males aged 25–59 years and females aged 15–59 years – Chókwè Health Demographic Surveillance System (Round 5), Chókwè District, Mozambique, April 2018–March 2019

Sex, circumcision status, and beliefs	% (95% CI)
**Males**	
**Circumcised**	
Underwent MC in the past year	3.0 (1.4–6.3)
Know that MC is partially protective against HIV infection	85.4 (80.5–90.5)
**Uncircumcised**	
Intend to undergo MC in the next year	44.7 (37.6–53.0)
Know that MC is partially protective against HIV infection	70.5 (63.4–78.5)
Reason for not undergoing circumcision*	
Any reason	>99.5% (NC)
Other^†^	55.5 (48.4–63.7)
Fear of complications^§^	26.6 (20.5–34.4)
Not part of my culture	17.2 (12.3–24.1)
Lack of time	17.0 (12.1–23.9)
Risk for injury to penis	13.3 (9.1–19.4)
Pain caused by procedure	9.3 (5.7–15.2)
Risk for infection	6.4 (3.5–11.4)
Does not prevent STI	1.9 (0.6–6.3)
Does not prevent HIV	1.9 (0.6–6.3)
Risk for impotence	0.6 (0.1–2.4)
Costs too much money	0.6 (0.1–2.4)
Sex is worse/less pleasurable	<0.5 (NC)
Partner does not want me to be circumcised	<0.5 (NC)
Looks unnatural	<0.5 (NC)
Risk for infertility	<0.5 (NC)
Contrary to my religious beliefs	<0.5 (NC)
**Females**	
Believe males should be circumcised	96.0 (95.1–96.9)
Ever discussed circumcision with a male sex partner or male friend or family member	29.2 (27.2–31.4)

** Abbreviations:** CI = confidence interval; MC = medical circumcision; NC = not calculated; R5 = round five; STI = sexually transmitted infection.

* Participants could indicate multiple reasons. All participants during R5 (April 2018–March 2019) indicated at least one reason why they did not undergo circumcision.

^†^ ”Other” reason for not undergoing circumcision was a free text field. No data existed for 50.5% of responses (unweighted). Of responses with data, lack of time was the most common reason for not undergoing circumcision (32.0% of unweighted data).

§ Composite variable combining risk for injury to penis, risk for infection, or pain caused by procedure.

HIV prevalence was lower among circumcised males than among uncircumcised males across all survey rounds (Figure). The age-adjusted difference in HIV prevalence between circumcised and uncircumcised males was significantly lower during R1–R4 (R1: HIV prevalence 12.7% versus 25.7% [aPR = 0.67; p = 0.005]; R2: HIV prevalence 10.5% versus 30.9% [aPR = 0.55; p = <0.001]; R3: HIV prevalence 9.6% versus 28.9% [aPR = 0.62; p = 0.002]; R4: HIV prevalence 11.2% versus 32.1% [aPR = 0.65; p = 0.005]). The pattern was similar during R5, but the difference was not statistically significant (R5: HIV prevalence 11.8% versus 27.3% [aPR = 0.81; p = 0.188]). During R1–R3, annual HIV incidence was 0.2% among circumcised males and 3.2% among uncircumcised males (incidence difference p = 0.02).

**FIGURE:**
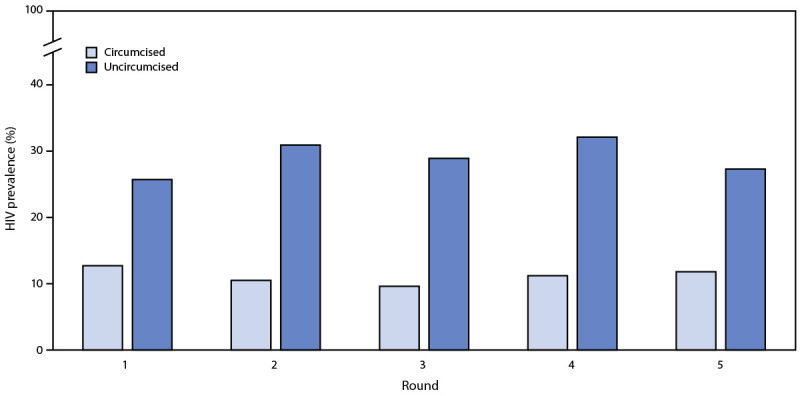
HIV prevalence among males aged 15–59 years, by circumcision status and survey round*^,^^†^ — Chókwè Health Demographic Surveillance System, Chókwè District, Mozambique, 2014–2019 **Abbreviations**: aPR = age-adjusted prevalence ratio; CI = confidence interval; R = round. * R1: April 2014–April 2015; R2: May 2015–January 2016; R3: March–December 2016; R4: March–November 2017; R5: April 2018–March 2019. † aPRs (95% CIs) were calculated by survey round: R1 = 0.67 (0.51–0.89); R2 = 0.55 (0.40–0.76); R3 = 0.62 (0.46–0.83); R4 = 0.65 (0.49–0.88); and R5 = 0.81 (0.60–1.11).

## Discussion

During 2014–2019, the prevalence of circumcision increased 42% among males aged 15–59 years in the Chókwè District of Mozambique, which has a high HIV prevalence, after implementation of a program to promote circumcision for HIV prevention. The prevalence of circumcision in 2019 was lower among males aged 25–59 years than among younger males and remains a critical gap because peak incidence of HIV in Mozambique occurs among males aged 35–39 years (*4*). For the VMMC program to exert its most immediate public health impact, males in the age group or groups with the highest HIV incidence need to become circumcised.

Circumcision prevalence did not reach the target of 80% among males aged 25–59 years despite a high proportion who stated an intent to become circumcised, indicating unaddressed barriers among these males. This analysis identified various barriers (e.g., fear of complications, not perceiving male circumcision as part of one’s culture, or lack of time), indicating that a multifaceted approach is needed to increase circumcision among these males. The VMMC program could address commonly reported barriers by expanding the availability of services through extended hours and additional community-based services, and by conducting campaigns specifically targeting males aged 25–59 years. The program should also address competing priorities that lead to lack of time (*5*), promote and ensure the safety of circumcision (*6*), and engage community leaders and other important influencers to promote circumcision (*5*). In addition, females, who nearly universally supported circumcision in the CHDSS, could be encouraged to promote circumcision with male sex partners, family members, and friends (*7*). Lastly, a knowledge gap among uncircumcised males of the partially protective benefit of male circumcision illustrates the continued need for education regarding the benefits of male circumcision in Chókwè District. However, the remaining gap in circumcision among males aged 25–59 years could result from a higher proportion being in monogamous sexual relationships compared with those aged 15–24 years and, consequently, a lower perceived need for the procedure.

As expected, HIV prevalence and incidence were lower among circumcised males than among uncircumcised males during R1–R5, even after adjusting for age. Although HIV prevalence was lower for circumcised males than uncircumcised males during R5, the difference was not statistically significant after adjusting for age. The lack of statistical significance might be attributed to an increasing proportion of older males, many of whom had HIV infections, undergoing circumcision, or higher mortality among HIV-positive males, more of whom were uncircumcised compared with HIV-negative males. Also, lower power to detect differences because of a smaller sample size of older males in R5 or self-misclassification of circumcision status by uncircumcised males related to a desire to align with perceived preference of CHDSS survey staff, especially as increasing proportion of males in Chókwè were circumcised, could contribute to this finding.

The findings in this report are subject to at least six limitations. First, these findings do not reflect trends among males aged 10–14 years, a group that accounted for approximately 50% of VMMC clients in Mozambique (*8*). Second, although annual surveys were based on a random sample of households and results were weighted to the census, the generalizability of these findings outside of the CHDSS in Chókwè District (or Mozambique) is unknown. Third, self-reported circumcision status can be unreliable (*9*), but it might be more accurate in areas where male circumcision is not a local cultural practice (*10*). Fourth, although a large proportion of uncircumcised males stated an intent to become circumcised, this could reflect a social desirability bias among some who had no intention of being circumcised. Fifth, because the study included few incident HIV infections, recency results needed to be pooled across R1–R3. Finally, differences in other HIV risk behaviors (e.g., number of sex partners) could account for the association of lower HIV prevalence with male circumcision.

This analysis demonstrates increasing prevalence of male circumcision in the context of VMMC program implementation. Reaching 90% circumcision prevalence among males aged 15–24 years in CHDSS is a notable achievement, which was attained with a circumcision program that involved routine and campaign VMMC service delivery, public engagement for demand creation, circumcision promotion by community health workers, and free transportation. Given the proven benefit of circumcision to reduce the risk for HIV infection, the lower prevalence among males aged 25–59 years in Chókwè District justifies continued promotion of VMMC services as a critical component of the HIV response in this hyperendemic area. Fear of complications, cultural reasons, and lack of time were among the most commonly reported reasons for not undergoing circumcision by males aged 25–59 years. A multifaceted strategy could address barriers to circumcision. These include reassuring the population that services are safe, engaging key influencers, providing convenient service delivery, addressing the competing priorities of males eligible for VMMC, and shifting social norms.

**Summary**

**What is already known about this topic?**

Circumcision reduces the risk for heterosexually acquired HIV infection among males and is an important HIV-preventive strategy in Mozambique. Voluntary medical male circumcision programs have been supported by the Mozambique Ministry of Health since 2009.

**What is added by this report?**

During 2014–2019, the prevalence of male circumcision increased 42% in Chókwè District in southern Mozambique. The largest increase occurred among males aged 15–24 years; the prevalence among those 25–59 years remained below the national objective of 80%. Fear of complications, cultural reasons, and lack of time were among the most common reasons reported for not undergoing circumcision by males aged 25–59 years.

**What are the implications for public health practice?**

A multifaceted strategy addressing concerns about the safety of the procedure, cultural norms, and competing priorities could help overcome barriers to circumcision among males aged ≥25 years.

## Footnotes

* Deceased.

† Program performance data were obtained from Jhpiego (a nonprofit affiliate of Johns Hopkins University), which is a VMMC service provider.

§ Survey dates were as follows: R1 = April 2014–April 2015, R2 = May 2015–January 2016, R3 = March–December 2016, R4 = March–November 2017, and R5 = April 2018–March 2019.

¶ Assessed recency of HIV infection with a limiting-antigen (LAg) avidity enzyme immunoassay using dried blood spots. LAg-avidity tests were not performed on specimens during R4 and R5. https://www.maximbio.com/Products/92003/Maxim-HIV-1-Limiting-Antigen-Avidity-%28LAg-Avidity%29-DBS-EIA-Kit%2C-192-Tests

** Nonsingle was a composite variable of married, union, divorced, separated, and widowed.

†† Recency test results were used to calculate annualized HIV incidence. Participants who reported using antiretroviral therapy or who had HIV viral load suppression (<1000 copies/mL) were defined as having long-term infection. Participants with recent infection were assumed to have a mean duration of recent infection of 161 days. Analysis was conducted using R Incidence Estimation Tools package. https://github.com/SACEMA/inctools

§§ Data across R1–R3 were pooled because of eight recent infections that occurred after repeat participants were excluded.

¶¶ 45 C.F.R. part 46.102(l)(2), 21 C.F.R. part 56; 42 U.S.C. Sect. 241(d); 5 U.S.C. Sect. 552a; 44 U.S.C. Sect. 3501 et seq.

*** A composite variable for fear of complications included risk for injury to penis, risk for infection, and pain caused by the procedure.

††† R1–R4 with a similar proportion.
